# Ubiquinol supplementation in elderly patients undergoing aortic valve replacement: biochemical and clinical aspects

**DOI:** 10.18632/aging.103742

**Published:** 2020-07-31

**Authors:** Patrick Orlando, Jacopo Sabbatinelli, Sonia Silvestri, Fabio Marcheggiani, Ilenia Cirilli, Phiwayinkosi Vusi Dludla, Alberto Molardi, Francesco Nicolini, Luca Tiano

**Affiliations:** 1 Department of Life and Environmental Sciences, Università Politecnica delle Marche, Via Brecce Bianche, Ancona 60100, Italy; 2 Department of Clinical and Molecular Sciences, DISCLIMO, Università Politecnica delle Marche, Ancona 60100, Italy; 3 Biomedical Research and Innovation Platform, South African Medical Research Council, Tygerberg 7505, South Africa; 4 Cardiac Surgery Department, Parma University Hospital, Parma 43126, Italy

**Keywords:** ubiquinol, cardiac surgery, ageing, oxidative stress

## Abstract

Epidemiological data show a rise in the mean age of patients affected by heart disease undergoing cardiac surgery. Senescent myocardium reduces the tolerance to ischemic stress and there are indications about age-associated deficit in post-operative cardiac performance. Coenzyme Q10 (CoQ10), and more specifically its reduced form ubiquinol (QH), improve several conditions related to bioenergetic deficit or increased exposure to oxidative stress. This trial (Eudra-CT 2009-015826-13) evaluated the clinical and biochemical effects of ubiquinol in 50 elderly patients affected by severe aortic stenosis undergoing aortic valve replacement and randomized to either placebo or 400 mg/day ubiquinol from 7 days before to 5 days after surgery. Plasma and cardiac tissue CoQ10 levels and oxidative status, circulating troponin I, CK-MB (primary endpoints), IL-6 and S100B were assessed. Moreover, main cardiac adverse effects, NYHA class, contractility and myocardial hypertrophy (secondary endpoints) were evaluated during a 6-month follow-up visit. Ubiquinol treatment counteracted the post-operative plasma CoQ10 decline (p<0.0001) and oxidation (p=0.038) and curbed the post-operative increase in troponin I (QH, 1.90 [1.47–2.48] ng/dL; placebo, 4.03 [2.45–6.63] ng/dL; p=0.007) related to cardiac surgery. Moreover, ubiquinol prevented the adverse outcomes that might have been associated with defective left ventricular ejection fraction recovery in elderly patients.

## INTRODUCTION

Aortic stenosis (AS) is a chronic progressive disease that begins with sclerosis and progresses to more severe calcification of a trileaflet valve which causes a significant obstruction hampering the ejection from the left ventricle. Long considered as a passive degenerative process, AS shares in its complex pathobiology a number of risk factors with other atherosclerotic processes, including male gender, diabetes, dyslipidemia (low-density lipoprotein cholesterol [LDL-C] and low levels of high-density lipoprotein cholesterol [HDL-C]), lipoprotein(a), metabolic syndrome and smoking [1–7]. AS represents the most common type of valve disease [8], as well as the most prevalent form of cardiovascular disease (CVD) in the Western world after hypertension and coronary artery disease [9]. The population at risk increases in proportion to the improvement in life expectancy and it is also likely that AS prevalence will progressively increase even further [10]. In fact, AS affects about 0.2% of adults aged 50-59, 1.3% of those aged 60-69 years, 3,9% of individuals aged 70-79 and 9.8% of adults over 80 [11]. In the absence of treatments to prevent or promote the regression of the disease, a surgical aortic valve replacement (AVR) is currently the only therapeutic option [12].

Although a growing body of evidence supports the success of isolated AVR in elderly patients [13–17], advanced age is still being considered as a major deterrent to AVR. Nonetheless, results from large multicenter randomized trials including octogenarians undergoing AVR showed adverse outcomes and 1-year mortality rates comparable to younger patients [18–20]. Based on these convincing data, the 2014 ACC/AHA guidelines did not include advanced age among the contraindications to AVR [20].

However, despite surgical and medical progress in myocardial protection during cardiopulmonary bypass (CPB), clear evidence support age-related deficits in myocardial performance after cardioplegic arrest and perioperative stress [21, 22]. In fact, myocardial ischemia and subsequent reperfusion are unavoidable in patients undergoing cardiac surgery and can induce myocardial damage and recovery impairment [23–25]. The CBP-related ischemia-reperfusion injury promotes the local and systemic release of proteases, cytokines and ROS by activated leukocytes, inducing myocardial oxidative stress and inflammation [26–28].

Furthermore, it is important to take into account that often subjects undergoing AVR are characterized by an altered oxidative status either caused by an age-related decline in endogenous antioxidant defense or coexisting morbidities such as diabetes, dyslipidemia, metabolic syndrome frequently occurring in these patients.

Due to the above-mentioned critical issues, optimal patient preparation, involving also supplementation with antioxidants and bioactive molecules, has been addressed as a useful strategy for improving the bioenergetic and metabolic state of the patient in order to counteract cardiac surgery-related side effects [29].

Among these compounds, Coenzyme Q_10_ (CoQ_10_) is a lipophilic endogenous quinone ubiquitous in biological membranes. CoQ_10_ plays a major role in cellular bioenergetics acting as an electron shuttle between mitochondrial respiratory complexes by cycling between its oxidized (ubiquinone) and reduced (ubiquinol) form. Accordingly, tissues characterized by high respiratory demand and energy turnover, such as the cardiac muscle, are particularly rich in CoQ_10_ [30].

Moreover, in its reduced form CoQ_10_ is also endowed with critical antioxidant properties in the lipid environment [31]. In particular, at the extracellular level CoQ_10_ is carried in plasma by lipoproteins where, in physiological conditions, it is present mainly (over 90%) in the ubiquinol form. Among lipoproteins, low-density lipoproteins (LDL) constitute the major carriers where ubiquinol, synergistically with vitamin E, plays a pivotal role in the inhibition of lipid peroxidation [30].

Observational studies have reported that plasma CoQ_10_ concentration is an independent predictor of mortality in patients with congestive heart failure [32]. Moreover, CoQ_10_ synthesis in humans progressively declines after 20 years of age [33], as well as the activities of the reductases responsible for CoQ activation to its reduced form [34]. These two observations highlight the relevance of age-associated CoQ_10_ deficit to enhanced cardiovascular risk.

In this context, the cardiovascular benefits of CoQ_10_ supplementation are among its best-documented clinical effects [35]. This is manly associated to its ability to protect LDL from oxidation and to counteract endothelial dysfunction also by modulating endogenous antioxidant defenses [36]. Moreover CoQ_10_, due to its bioenergetic role, was also shown to support myocardial contractility. In fact, CoQ_10_ supplementation was able to improve some cardiovascular indices, such as left ventricular ejection fraction (LVEF), cardiac output (CO) and stroke index (SI) [37]. Moreover, Belardinelli et al. [36] and Fotino et al. [38] demonstrated that the benefits of CoQ_10_ supplementation are more evident in patients with an EF > 30% or belonging to the II or III classes of New York Heart Association (NYHA). In cardiac surgery, CoQ_10_ supplementation enhanced mitochondria and myofilaments protection against oxidative stress, contributed to the maintenance of efficient energy production and improved contractile recovery after hypoxia-reoxygenation stress *in vitro* [29]. Taken together, such observations provide a strong rationale for CoQ_10_ supplementation in elderly subjects undergoing cardiac surgery.

In this context, the present study aimed to evaluate the role of oral supplementation with 400 mg/day ubiquinol in counteracting the oxidative and inflammatory effects related to aortic valve replacement surgery in elderly patients and in improving post-operative myocardial protection and systolic function.

## RESULTS

### Ubiquinol supplementation promotes a significant increase of total CoQ_10_ plasma levels after cardiac surgery protecting from oxidation

**Figure 1:**
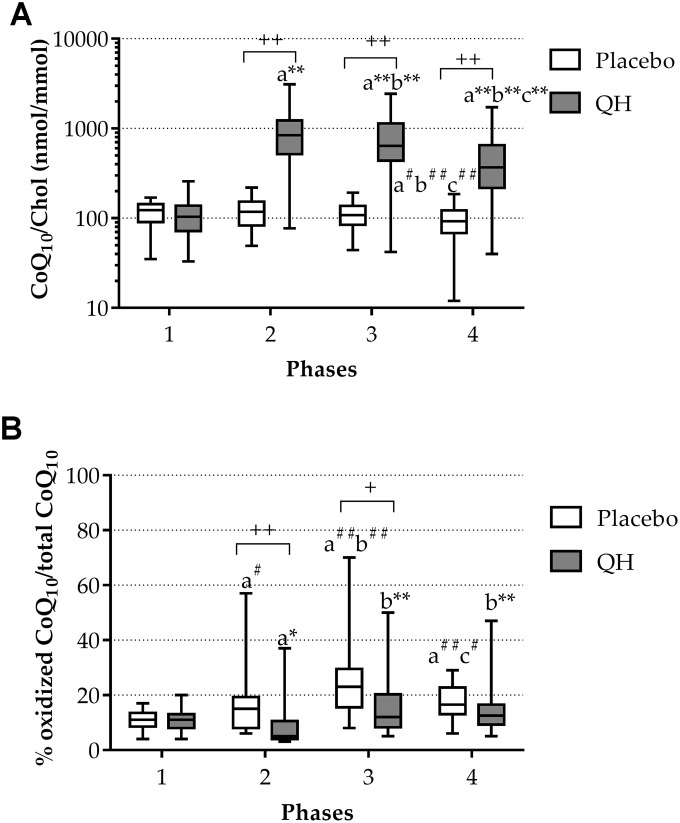
Total CoQ_10_ plasma levels normalized to cholesterol (**A**) and its oxidative status (**B**) in placebo (white) and QH treated (grey) groups during the four experimental phases. * p≤0.05, ** p≤0.01 and ^#^p≤0.05, ^##^p≤0.01 significance of differences in each subgroup compared with phase 1 (**a**), 2 (**b**) and 3 (**c**). + p≤0.05 and ++ p≤0.01 significance of differences comparing both groups at the same experimental phase.

At study entry, both treatment groups were homogeneous in terms of plasma total CoQ_10_ levels (QH treated = 110.34 ± 11.75 nmol CoQ_10_/mmol chol; placebo = 115.85 ± 6.92 nmol CoQ_10_/mmol chol; p=0.67) (Figure 1A) and its oxidative status (QH treated = 11 ± 1% CoQ_10_ ox/tot CoQ_10_; placebo = 11 ± 1% CoQ_10_ ox/tot CoQ_10_; p=0.764) (Figure 1B). Cardiac surgery procedure did not immediately modify CoQ_10_ status but a significant decrease of total plasma CoQ_10_ was observed in the placebo group compared to entry level at phase 4 (*i.e.* 5 days after the procedure).

Ubiquinol supplementation led to a highly significant increase of its plasma level in all experimental phases compared to baseline values (Figure 1A). In fact, although a progressive decline in total plasma CoQ_10_ content was observed during phases 3 and 4 also in the treated group (789.58 ± 120.53 nmol CoQ_10_/mmol chol and 491.58 ± 90.42 nmol CoQ_10_/mmol chol, respectively), CoQ_10_ content remained significantly higher in comparison with basal level and placebo group at all experimental points. Moreover, increased levels of plasma total CoQ_10_ efficiently counteracted its oxidation induced by the cardiac surgery procedure (Figure 1A). In fact, in ubiquinol supplemented patients, the percentage of the oxidized form decreased at the phase 2, despite the surgical procedure (phase 1 = 11 ± 1% CoQ_10_ ox/tot CoQ_10_; phase 2 = 8 ± 2% CoQ_10_ ox/tot CoQ_10_; p=0.05) and remained unchanged in the last 2 phases compared to the basal condition. On the contrary, in the placebo group, while total CoQ_10_ levels did not seem to be immediately affected by the procedure (Figure 1A), an increase in the percentage of oxidized CoQ_10_ following the cardiac surgery procedure occurred (Figure 1B). Figure 1B highlights the progressive increase in the percentage of ubiquinone on total CoQ_10_ in placebo patients at phases 2 and 3 (phase 2 = 14 ± 1% CoQ_10_ ox/tot CoQ_10_; phase 3 = 25 ± 3% CoQ_10_ ox/tot CoQ_10_), which was shown to occur at a significantly greater extent compared to the QH-treated group at the same experimental moments (p = 0.012 and p = 0.015, respectively). Table 1 summarizes CoQ_10_ plasma levels and oxidative status in the two study arms at each time point.

**Table 1:** Summary of the primary endpoints i) plasma and tissue CoQ10 levels, and ii) plasma levels of specific biomarkers of ischemia-reperfusion damage (Troponin I and CK-MB).

**Variable**	**QH (n=24)**	**Placebo (n=22)**	**P value**
**Plasma CoQ_10_ (nmol/mmol chol)**			
Phase 1	110.34 ± 11.75	115.85 ± 6.92	0.990
Phase 2	946.81 ± 149.63	120.31 ± 8.28	<0.001
Phase 3	789.58 ± 123.53	112.64 ± 7.48	<0.001
Phase 4	491.58 ± 90.42	94.67 ± 7.89	0.001
**Plasma oxidized CoQ_10_ (%)**			
Phase 1	11 ± 1	11 ± 1	0.764
Phase 2	8 ± 2	14 ± 1	0.012
Phase 3	16 ± 2	25 ± 3	0.015
Phase 4	15 ± 2	17 ± 1	0.312
**Cardiac CoQ_10_ content (μg/mg tissue)**	0.047 ± 0.004	0.047 ± 0.003	0.973
**Heart oxidized CoQ_10_ (%)**	67 ± 2	70 ± 2	0.339
**Troponin I (ng/dL)**			
Phase 1	0.02 (0.01 – 0.03)	0.06 (0.03 – 0.16)	0.098
Phase 2	2.67 (1.90 – 3.75)	2.92 (2.10 – 4.06)	0.992
Phase 3	1.90 (1.47 – 2.48)	4.03 (2.45 – 6.63)	0.007
Phase 4	0.29 (0.14 – 0.63)	0.49 (0.23 – 1.02)	0.783
**CK-MB (ng/mL)**			
Phase 1	1.35 ± 0.14	2.42 ± 0.58	0.343
Phase 2	30.10 ± 7.32	31.21 ± 4.56	0.999
Phase 3	18.34 ± 2.47	32.93 ± 11.28	0.195
Phase 4	2.53 ± 0.52	5.86 ± 1.00	0.023

### Cardiac CoQ_10_ levels and its oxidative status was not different in both experimental groups

**Figure 2:**
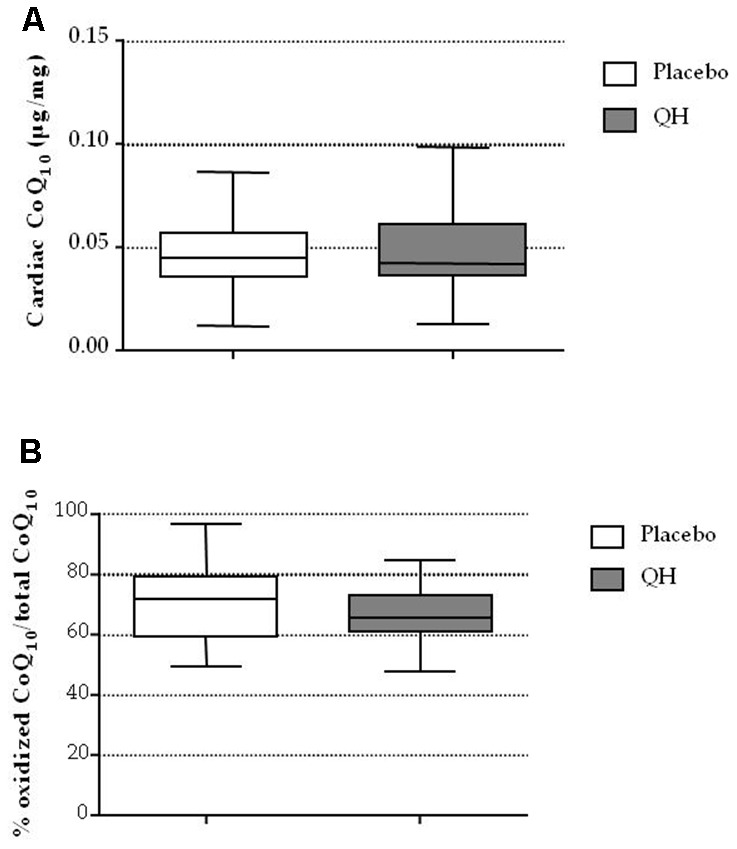
Cardiac CoQ_10_ levels (**A**) and its oxidative status (**B**) in placebo (white) and QH treated (grey) groups.

As shown in Figure 2 and in Table 1, ubiquinol supplementation did not affect total CoQ_10_ content nor its oxidative status in cardiac tissue of patients. In fact, no significant difference between experimental groups was recorded in terms of cardiac CoQ_10_ content (QH treated = 0.047 ± 0.004 μg CoQ_10_/mg cardiac tissue; placebo = 0.047 ± 0.003 μg CoQ_10_/mg cardiac tissue; p=0.973) and oxidized CoQ_10_ (QH treated = 67 ± 2% CoQ_10_ ox/tot CoQ_10_; placebo = 70 ± 2% CoQ_10_ ox/tot CoQ_10_; p=0.339).

### Ubiquinol supplementation did not prevent cardiac surgery-induced increases in plasma IL-6 and S100B protein levels

**Figure 3:**
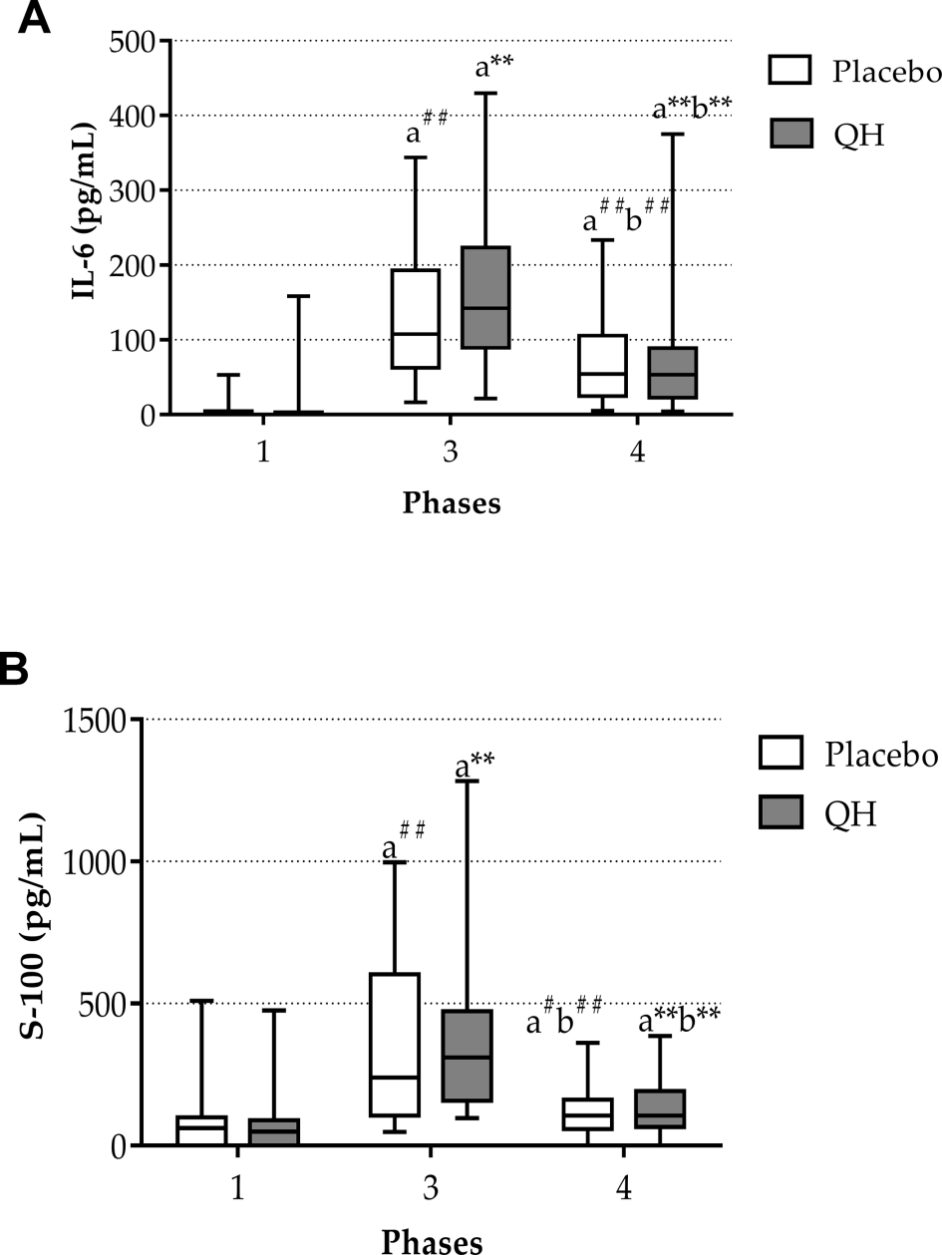
IL-6 (**A**) and S-100 protein (**B**) plasma levels in placebo (white) and QH treated patients (grey) at phase 1, 3 and 4. ** p≤0.01, ^#^p≤0.05 and ^##^p≤0.01 significance of differences in each experimental group in comparison with phase 1 (**a**) and 3 (**b**).

Plasma interleukin-6 (IL-6) and S100B protein levels were quantified in phases 1, 3 and 4 as markers of inflammation and cerebral damage, respectively. Figure 3 highlights that aortic valve replacement procedure led to a highly significant increase of both markers in both experimental groups. Indeed, immediately after surgery, plasma IL-6 levels raised from 5.1 ± 2.25 pg/mL to 137.1 ± 20.68 pg/mL (p<0.01) in placebo group and from 9.7 ± 7.87 pg/mL to 169.5 ± 26.21 pg/mL (p<0.01) in QH treated group. Similarly, also S100B protein levels increased from 66.5 ± 20.63 pg/mL to 368.7 ± 58.23 pg/mL (p<0.01) in placebo group and from 71.5 ± 24 pg/mL to 390.2 ± 66.77 pg/mL in QH treated group (p<0.01). However, at discharge, a significant decrease in all patients was observed, although levels remained elevated in comparison to study entry.

### Ubiquinol supplementation improved myocardial protection by counteracting plasma Troponin I and CK-MB post-surgery increase

**Figure 4:**
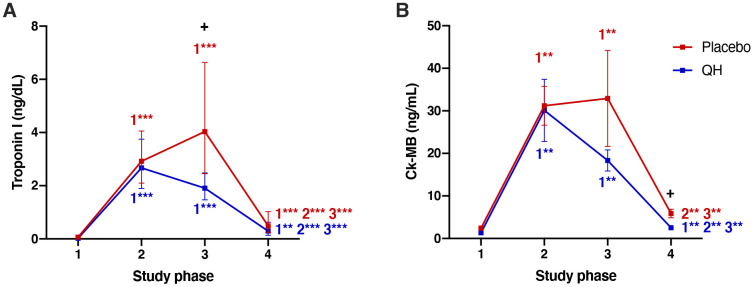
Troponin I (**A**) and Ck-MB (**B**) plasma levels in placebo (red) and QH treated (blue) groups during the four experimental phases. **p<0.01 and ***p<0.01 significance of differences in each experimental group in comparison with phase 1, 2 and 3 (c). + p<0.05 significance of differences comparing both groups at the same experimental phase.

Myocardial protection was evaluated by quantifying plasma Troponin I and CK-MB levels. As reported in Figure 4 and in Table 1, cardiac surgery caused an increase of both markers in all post-operative phases. However, the trends of both enzymes in placebo and QH treated groups were similar with the exception of data recorded 24 hours after surgery (phase 3), in which patients treated with ubiquinol showed significantly lower levels of troponin I in plasma compared to the placebo group (QH treated, 1.90 (1.47 – 2.48) ng/dL plasma; placebo, 4.03 (2.45 – 6.63) ng/dL; p=0.007). Moreover, ubiquinol supplementation was also able to curb the increase of plasmatic CK-MB although, in this case, differences compared to the placebo group were statistically significant at discharge, *i.e.* 5 days after the procedure (QH treated = 2.53 ± 0.52 ng/mL; placebo = 5.86 ± 1.00 ng/mL; p = 0.023), and not 24 hours after surgery (p = 0.195). Finally, both parameters were significantly lowered in plasma at discharge, but still significantly higher levels were observed in comparison to baseline values.

### NYHA class was similar in both experimental groups in the follow-up

**Figure 5:**
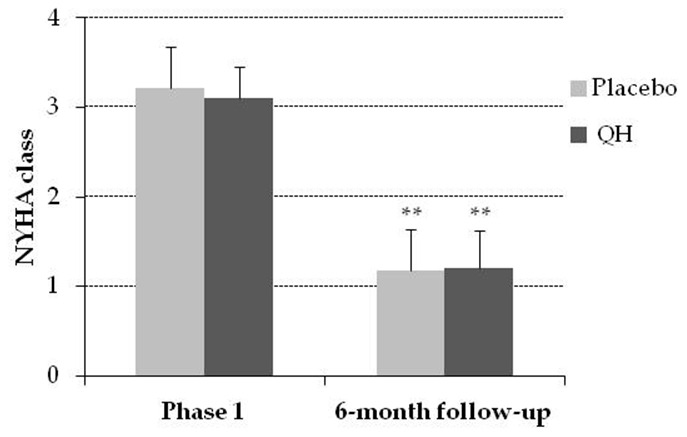
**NYHA class in placebo (grey) and QH treated (black) group in phase 1 and following 6-month follow-up.** **p≤0.01 significance of differences in each experimental group in comparison with phase 1.

Cardiac valve replacement significantly improved functional parameters related to heart failure quantified by means of NYHA classification 6 months after surgery in both experimental groups, as shown in Figure 5. However, ubiquinol treated patients did not show any significant additional improvement compared to the placebo group (NYHA class went from 3.17 ± 0.12 to 1.21 ± 0.10, in QH treated patients and from 3.23 ± 0.11 to 1.18 ± 0.08, p <0.01).

### Ubiquinol supplementation significantly improved systolic function in the follow-up phase

**Figure 6:**
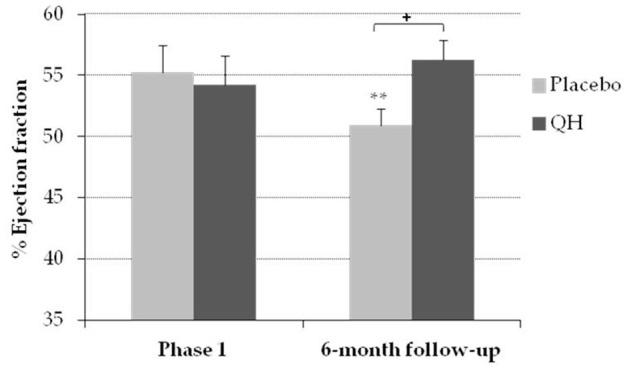
**Percentage of ejection fraction in placebo (grey) and QH treated (black) group in phase 1 and following 6-month follow-up.** **p≤0.01 significance of differences in each experimental group in comparison with phase 1; + p≤0.05 significance of differences between both groups in the follow-up.

LVEF was measured by echocardiography at study entry (phase 1) and during a 6-month follow up visit, in order to evaluate the left ventricular systolic function (Figure 6). At baseline, LVEF resulted homogeneous in both experimental groups (QH treated = 54% ± 2.42; placebo = 55% ± 2.23, p = 0.75), while in the follow-up period patients treated with ubiquinol showed an improved systolic function compared to placebo group (QH treated = 56% ± 1.65, placebo = 51% ± 1.38; p = 0.018).

### Frequency of major cardiac or systemic adverse events was not influenced by ubiquinol supplementation

Finally, by monitoring the patients for adverse events during the whole study duration, a low rate of adverse events was recorded, with the minor exception of atrial fibrillation that interested the 35% of the total population. No significant differences were observed between placebo and ubiquinol treated patients in the incidence of such events (Table 2).

**Table 2:** Complications related to cardiac surgery in QH treated and placebo groups.

**Complication**	**QH**	**Placebo**	**P value**
Prolonged Ventilation	0/24	0/22	-
Atrial Fibrillation	6/24	10/22	0.307
Complete AV Block	1/24	0/22	0.303
Major Arrhythmias	0/24	0/22	-
Acute Renal Failure	2/24	1/22	0.504
Dialysis	0/24	0/22	-
GI Complications	1/24	0/22	0.293
Stroke	0/24	0/22	-
Death	0/24	0/22	-

## DISCUSSION

Aortic Valve Replacement is an invasive procedure requiring open-heart surgery. Moreover, the intervention is largely conducted on elderly patients characterized by constitutive low-grade inflammation and imbalanced oxidative status typically associated with the senescence process, representing a further challenge in cardiac surgery. In this respect, supplementation with nutraceuticals to support endogenous defenses may represent a useful tool to prepare the elderly patients for this stressful procedure, as recently concluded in a meta-analysis [39].

Among nutraceuticals, Coenzyme Q10 cardioprotective role is well documented in terms of improved ejection fraction, cardiac output, cardiac index and telediastolic volume [37, 38, 40]. Moreover, myocardial CoQ_10_ deficiency in the majority of patients with AS or aortic insufficiency are known since the pioneering work of Karl Folkers in 1970 [41]. The present study aimed to evaluate the effect of ubiquinol, the reduced and active form of CoQ_10_, in improving biochemical and clinical parameters in elderly patients undergoing AVR. The choice of ubiquinol for this study, in addition to the proven enhanced bioavailability of the reduced form of CoQ_10_, is particularly tailored on aged individuals endowed with a lower activity of the reductases responsible for the activation of CoQ_10_ in comparison to younger individuals [35].

Surgical intervention, as expected, was able to alter the oxidative status and inflammatory response in patients undergoing AVR procedure. In particular, an increase in the percentage of oxidized CoQ_10_/total CoQ_10_ was evident 24 hours after cardiac surgery (phase 3). In this respect, ubiquinol supplementation efficiently counteracted plasma CoQ oxidation, which constitutes a sensitive marker of oxidative stress *in vivo* [42–45]. This result is likely due to ubiquinol antioxidant activity combined to its enhanced bioavailability, thus enabling to reach high plasma levels after only seven days of supplementation. Notably, mean perioperative plasma CoQ_10_ levels were greater than the 500 μmol/mol cholesterol threshold assumed to confer a significant benefit on CVD patients [46], demonstrating the effectiveness of the adopted dosage regimen (dose, schedule and duration of the ubiquinol supplementation).

Nonetheless, ubiquinol supplementation did not affect total CoQ_10_ content and its oxidative status in cardiac tissue. In fact, tissue bioavailability of exogenous CoQ_10_, particularly in cardiac and skeletal muscle is a debated topic. Several animal studies have shown that CoQ_10_ accumulates in some organs, particularly in the liver [47–49], but to a much lower extent in the heart, regardless of concentration [49] and time [48] of administration. Reahal demonstrated that also different routes of CoQ_10_ administration (oral and intraperitoneal) in rats did not impact its myocardial accumulation [47]. On the contrary, Kwong et al. reported an increase of CoQ_9_ (*i.e.* the major endogenous CoQ form in rodents) in whole homogenates from heart and in mitochondrial fraction following 4 weeks of treatment with 150 mg/kg/day CoQ_10_ in rats [50]. Probably, the accumulation of CoQ_10_ into the heart, differently from other organs, is limited and triggered by specific physiological conditions like increased energy demand, CoQ_10_ deficiency, or increased mitochondrial biogenesis. In fact, our recent study showed an increase of CoQ_9_ and CoQ_10_ accumulation in cardiac tissue of SAMP8 mice only when supplementation was associated with physical activity, suggesting an exercise-associated increase in CoQ request highlighted by a positive modulation of endogenous CoQ_9_ synthesis [51].

In contrast with our report, Rosenfeldt et al. observed a significant myocardial uptake of ubiquinone in patients undergoing cardiac surgery after 2 weeks of presurgical supplementation with 300 mg/day ubiquinone [29]. However, cardiac CoQ_10_ levels recorded in placebo patients of that study (17.2 μg/g wet weight tissue) was lower than the concentration usually reported in the literature. In fact, Ernster and Dallner measured an average of 47.2 μg CoQ_10_ per g of wet weight heart tissue in same-aged, not supplemented patients [52], which is in line with our data.

These discrepancies might reflect different characteristics of the patient cohort, since CoQ_10_ deficiency in the heart is proportional to the severity of the disease [53], and this may influence the bioavailability of exogenous CoQ at the tissue level.

Inflammation is known to occur in the post-cardiac surgery phase in association to trauma, abnormal shear stress, ischemia, reperfusion and hypothermia; as a result IL-6 plasma levels remain elevated in the post-surgery phase [54–59]. Recent studies highlighted a significant role of CoQ_10_ in counteracting the inflammatory response in biological systems by modulating the transcription of genes governing the proinflammatory JAK/STAT signaling pathways, by suppressing the activation of nuclear factor-κB (NF-κB) [60, 61], and by activating the peroxisome proliferator-activated receptor-mediated anti-inflammatory responses [62, 63].

However, in the present study ubiquinol supplementation was not able to able to curb the inflammatory response triggered by cardiac surgery procedure. Lack of anti-inflammatory response in our experimental setting might be due to a remarkably high inflammatory state (plasma IL-6 >100 pg/ml) associated with the post-surgical intervention. In fact, data in the literature supporting anti-inflammatory effect of CoQ_10_ *in vivo* where associated to lower levels of inflammation [64, 65].

Similarly, treatment with 400 mg/day ubiquinol was not able to limit the increase of plasma S100B protein, a known marker of soft tissue damage. S100B elevation is frequently associated with minor brain ischemia, which is relatively frequent in elderly patients undergoing AVR and recognized as biomarker of poor prognosis in these patients [66].

Nonetheless, our data highlighted a significant protective effect of ubiquinol during the surgical procedure by counteracting troponin I and Ck-MB plasma level increases, which is a major novelty in terms of ubiquinol myocardial protective role. Previous studies in cardiac surgery patients involving CoQ_10_ in the ubiquinone form where not able to highlight significant differences in these parameters [29, 67, 68]. In particular, while in previous studies different dosage and treatment protocols were used, the single most important difference in the present study is likely to be the use of the reduced and active form of CoQ_10_ that is known to be associated with an improved bioavailability, as shown both in animal [69] and human [70, 71] studies.

Patients in the placebo arm showed a significant decline in LVEF assessed 6 months after cardiac surgery, whereas no change in LVEF was observed in ubiquinol supplemented patients at follow-up visit. This result is consistent with previous reports showing that AVR does not induce a significant LVEF increase in patients with a normal preoperative EF [72, 73]. On the contrary, similarly to our experience, a slight decline in LVEF was observed following the surgical procedure. The mechanisms underlying the different responses in terms of LVEF recovery after AVR observed in patients with different preoperative LVEF are still debated. It has been hypothesized that in subjects with impaired LVEF, the elimination of the gradient across the aortic valve triggers a remodeling of the left ventricle that drives EF normalization [73]. Notably, ubiquinol supplementation prevented a postoperative decline in LVEF, which is associated with a higher risk of adverse outcomes, including major adverse cardiac and cerebrovascular events [74]. This effect could be related to the protective effect of ubiquinol against perioperative myocardial injury which was observed in the active arm of the study.

The beneficial effects of CoQ_10_ supplementation on systolic function have been previously demonstrated in patients with heart failure with reduced ejection fraction (HFrEF). Specifically, a metanalysis by Fotino et al. demonstrated a significant effect of CoQ_10_ in improving LVEF in patients with heart failure (basal % of ejection fraction exceeding 30%) (38). Similarly, the Q SYMBIO study [75], 300 mg/day of CoQ_10_ for 16 weeks were able to significantly improve NYHA class in chronic heart failure patients. In the present study, we report for the first time a protective effect of ubiquinol against the post-operative systolic dysfunction related to aortic valve replacement in a cohort of subjects presenting with heart failure and preserved ejection fraction (HFpEF).

Moreover, despite a significant difference in LVEF at the 6-month follow-up was observed in ubiquinol treated patients compared to placebo, both groups underwent a significant improvement in NYHA class, and no substantial additional improvement was observed in ubiquinol treated patients. This result can be explained by the clinical profile of the patients that was characterized by normal LVEF at study entry.

In conclusion, the study shows that, although AVR is considered a safe operation, it promotes oxidative stress, inflammation, tissue damage. Notably, the study highlights a significant decrease in plasma CoQ_10_ content in the days following surgery that could suggest an increased request by the tissue. Such decrease was efficiently prevented by ubiquinol supplementation. Notably, ubiquinol has been shown to be a useful antioxidant for myocardial protection by minimizing acute myocardial stress during intervention, assessed in terms of circulating troponin I and CK-MB, as well as in the prevention of the adverse outcomes related to a defective LVEF recovery in elderly patients.

## MATERIALS AND METHODS

### Experimental design

This monocentric, double blinded and randomized study was realized in collaboration with University Hospital of Parma, section of Cardiac Surgery. The study was approved by Parma’s Ethical Committee (Eudra CT number 2009-015826-13) and was conducted in accordance with the ethical principles of the Declaration of Helsinki. Informed consent was obtained from all study participants.

Fifty patients were enrolled for the present study between July 2010 and March 2014. Inclusion criteria were: age > 70 years old, diagnosis of Severe Aortic Stenosis (associated mild or moderate regurgitation were included) with surgical indication demonstrated by echocardiographic examination and cardiac catheterization. Exclusion Criteria were: urgency procedures following clinical emergency, acute myocardial infarction, re-intervention, other types of valve surgery, endocarditis and medical history positive for cancer in the past 5 years.

At the beginning of the study patients were randomized in two homogeneous groups: 25 of them were supplemented with ubiquinol 200 mg *bid* (QH absorb Jarrow Formulas®) (QH treated), while the remaining 25 were administrated placebo (soya lecithin and medium chain triglyceride), once a day with one of the main meals. Oral supplementation started 7 days before and ended 5 days after surgery. Treatment was discontinued only the day of surgery.

**Figure 7:**
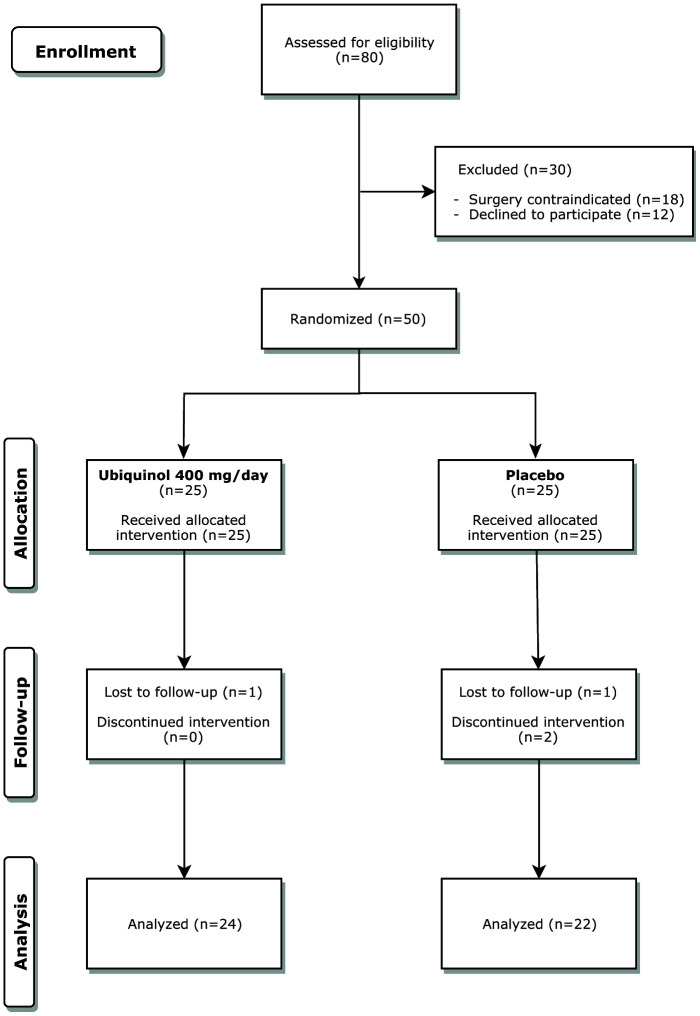
**CONSORT flow chart.** A total number of 80 patients were screened. Of these 50 were randomized to the three groups and 46 completed the study.

Four participants dropped out. The recruitment process and the reasons for withdrawal are summarized in the CONSORT flow chart in Figure 7. In line with the protocol, dropouts were not replaced. The analysed population therefore consists of 46 subjects, 24 in the QH group, and 22 in the placebo group. Their baseline demographic, clinical, and biochemical characteristics are reported in Table 3.

**Table 3:** Clinical and biochemical variables of the enrolled patients.

**Variables**		**QH (n=24)**	**Placebo (n=22)**
Males (n°)		12	12
Females (n°)		12	10
Age (years)		77.67±4.63	78.00±3.98
BMI (kg/m^2^)		26.45±5.41	26.70±3.29
BSA (m^2^)		1.77±0.18	1.80±0.18
Hypertension (n°)		21	20
Diabetes (n°)		3	5
Aethiology AVD (n°)	Degenerative	23	21
	Congenital	1	1
Central Neurological Dysfunction (n°)		0	1
Extracardiac Arteriopathy (n°)		6	2
COPD (n°)		3	3
Previous PTCA (n°)		4	3
AMI (n°)		1	2
Congestive Heart Failure (n°)		3	1
NYHA class (n°)	I	0	0
	II	2	1
	III	16	15
	IV	6	6
CABG associated (n°)		2	5
Euroscore (%)		7.08±1.86	7.55±1.60

**Figure 8:**
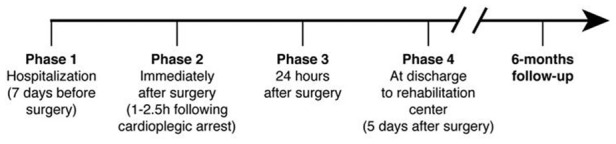
**Flow chart of study design.**

The study flow chart is reported in Figure 8 and consisted of 4 phases. At each phase blood was withdrawn from a peripheral vein into lithium-heparin vacutainers. Subsequently plasma, obtained by centrifugation at 1,600 x g for 5 minutes, was immediately cryopreserved at -80°C until used to evaluate CoQ_10_ content and oxidative status as well as Interleukin-6 (IL-6), S100B protein, Troponin I (TnI), creatine phosphokinase-MB (CK-MB) levels. Moreover, NYHA class and the ejection fraction of left ventricles was assessed at discharge from hospital and during the 6 months follow-up visit. Major Cardiac or Systemic adverse events during hospitalization or during follow up were recorded.

### Primary and secondary endpoints

The primary endpoints of the study were: i) the evaluation of plasma and tissue CoQ_10_ levels at Phases 2 and 3, and ii) level of intraoperative myocardial protection, assessed in terms of incidence of major ventricular arrythmias and plasma levels of specific biomarkers of ischemia-reperfusion damage (Troponin I and CK-MB). Secondary endpoints were: i) incidence of major cardiac adverse events in the 6-months follow-up; ii) heart failure symptoms and quality of life at 6 months after surgery, assessed by NYHA functional class; iii) analysis of cardiac function at 6 months after surgery, assessed by ejection fraction (EF).

### Surgical Technique

The aortic valve replacement was performed by three consultant cardiac surgeons through open-heart surgery in all patients. Median complete sternotomy, heparin subministation protocols, cannulation sites and vent insertion site were the same for all patients but we left the surgeons free to decide the type of cardioplegic solution to adopt (haematic or crystalloid). Extracorporeal circulation (ECC) was routinely used. Moreover, surgeons were left free to decide which type of aortic prosthesis to use. Before the cannulation of the right atrium an auricle biopsy was drawn and immediately kept at -80°C in order to quantify CoQ_10_ level and its oxidative status in cardiac tissue.

### Coenzyme Q_10_ levels and its oxidative status

Total CoQ_10_ content and its oxidative status were assayed in plasma [76] and cardiac tissue [51] by using a HPLC system. The extraction and the quantification steps were optimized in order to minimize the oxidation of samples due to the methodological procedures. In particular, plasma was thawed at 4°C and CoQ_10_ was extracted following a single dilution step by adding 250 μL propanol to 50 μL of plasma on ice followed by vigorous mixing on a vortex.

Meanwhile, in order to extract CoQ_10_ from the atrial tissue, biopsies were weighted and lysed using TissueLyser II (Qiagen) through 2 cycles of 2 minutes at 30 Hertz following addition of one mL propanol previously refrigerated and one stainless steel bead (7 mm of diameter, Qiagen). After extraction steps, plasma and tissue samples were centrifuged at 20,900 x g for 2 minutes at 4°C and 40 μL of supernatant was loaded in HPLC system using a refrigerated autosampler that guarantees optimal storage of the sample and minimizes oxidation also during the analytical phase. Notably, using this single dilution step extraction CoQ10 oxidation in the sample is marginal. Plasma CoQ_10_ levels were normalized by total cholesterol content (nmol CoQ_10_/mmol Chol), while its content in atrial biopsy was expressed as μg CoQ_10_/mg tissue. Levels of oxidation were expressed as percentage of ubiquinone/total CoQ_10_.

### Interleukin-6

Plasma IL-6 level was measured through ELISA test using a biotin-conjugated anti-Human IL-6 as first antibody and Streptavidin HRP as second one. The assay was performed according to the custom protocol (Human Interleukin-6 ELISA kit, BioVendor®) and the results were expressed as pg IL-6/mL plasma.

### S100B protein

S-100B protein was measured in plasma by a sandwich ELISA using a polyclonal anti-cow S100B antibody. The assay was performed according to the custom protocol (Human S100B ELISA kit, BioVendor®) and the results were expressed as pg S100B/mL plasma.

### Troponin I and CPK-MB

Troponin I and CPK-MB values were measured in plasma by chemiluminescence method using a DXI 800 (Beckman Coulter, Georgia, US). Results were expressed as ng troponin I/dL plasma and ng CPK-MB/dL plasma**.**

### Ejection Fraction

Ejection fraction was evaluated by means echocardiographic analysis and it is calculated by dividing the volume of blood pumped from the left ventricle per beat by the volume of blood collected in the left ventricle at the end of diastolic filling. Results were expressed as percentage.

### Major Cardiac or Systemic adverse events

The major cardiac or systemic adverse events considered were: Prolonged ventilation (>48 hours), New onset of Atrial Fibrillation, Major arrhythmias (FV, TV), Complete atrioventricular block requiring pacemaker implantation, Perioperative Myocardial infarction, Acute Renal Failure (requiring or not dyalysis), Gastrointestinal complications (Alitiasic Cholecystitis, Bowel ischemia), Cerebral Stroke, Death.

### Sample size and statistical analysis

Sample size calculation with respect to the primary endpoint indicated that a total of 42 patients would be required to detect a between-groups difference of 2 ng/dL in 24-hour post-operative plasma TnI levels, with a two-sided significance level of 0.05 and a power of 80%. Considering an anticipated dropout rate of 20%, a total of 50 patients was deemed as appropriate. Mean value, standard deviation and standard error of means (SEM) were calculated. All values were presented as means ± SEM. All statistical analyses were performed using GraphPad Prism^®^ 8.2 Software. TnI was log transformed prior to analysis and data presented as geometric mean with 95% CI. Unpaired two-tailed t-test was employed to compare placebo and QH groups and one-way repeated measures ANOVA with Greenhouse-Geisser correction to compare all the phases of study in each experimental group. In this case, post-hoc analysis of differences between phases was calculated using Tukey’s significant differences method. P≤0.05 and p≤0.01 were considered statistically significant and highly significant, respectively. Data were represented with histograms, line charts and box plot where mean, median and quartile values were reported. The central line and the box represent respectively the median, 75% and 25% of the measurements for each measurement.
